# *In Vitro* Studies of Polyhedral Oligo Silsesquioxanes: Evidence for Their Low Cytotoxicity

**DOI:** 10.3390/ma8095291

**Published:** 2015-09-10

**Authors:** Anna Janaszewska, Kinga Gradzinska, Monika Marcinkowska, Barbara Klajnert-Maculewicz, Wlodzimierz A. Stanczyk

**Affiliations:** 1 Department of General Biophysics, Faculty of Biology and Environmental Protection, University of Lodz, Pomorska 141/143, Lodz 90-236, Poland; E-Mails: ankuj@poczta.onet.pl (A.J.); m_monika123@interia.eu (M.M.); aklajn@biol.uni.lodz.pl (B.K.-M.); 2 Centre of Molecular and Macromolecular Sciences, Sienkiewicza 112, Lodz 90-236, Poland; E-Mail: kgradzin@cbmm.lodz.pl; 3 Leibniz-Institut für Polymerforschung Dresden e.V., Hohe Strasse 6, Dresden 01069, Germany

**Keywords:** POSS, silsesquioxane, *in vitro* toxicity, MTT assay, cell cycle, apoptosis, necrosis, reactive oxygen species

## Abstract

As scientific literature considers polyhedral oligosilsesquioxanes (POSS) as potential drug delivery systems, it is necessary to check their impact on mammalian cells. Toxicity of octaammonium chloride salt of octaaminopropyl polyhedral oligomeric silsesquioxane (oap-POSS) towards two cell lines: mouse neuroblastoma (N2a) and embryonic mouse hippocampal cells (mHippoE-18) was studied. Experiments consisted of analysis of a cell cycle, cell viability, amount of apoptotic and necrotic cells, and generation of reactive oxygen species (ROS). POSS caused a shift in the cell population from the S and M/G_2_ phases to the G_0_/G_1_ phase. However, the changes affected less than 10% of the cell population and were not accompanied by increased cytotoxicity. POSS did not induce either apoptosis or necrosis and did not generate reactive oxygen species. A cytotoxicity profile of POSS makes it a promising starting material as drug carrier.

## 1. Introduction

An intense search for new transporting systems of anti-cancer drugs is urgently needed. It is believed that a targeted therapy can both diminish severe side-effects and increase effectiveness of chemotherapy. Such a therapy requires combination of targeted molecules (e.g., folic acid residues) and chemotherapeutics. This can be achieved thanks to multivalency that characterizes some nanosystems such as dendrimers [1,2] or hyperbranched polymers [3,4], which possess plenty of functional groups. Despite promising results achieved for these polymers, there is still a point to look for simpler, more effective, and cheaper solutions.

Although silsesquioxanes were synthesized much earlier than other popular nanosystems, they are now regarded as a next generation materials in terms of their potential effectiveness in biomedicine as targeted carriers of anticancer drugs. Studies on polyhedral oligo silsesquioxanes (POSS) were limited to conjugation with fluorescent dyes [5], incorporation into dendrimeric systems [6,7], development of dental nano-composites [8] and cardiovascular implants [9]. Silsesquioxanes were scarcely studied as drug delivery systems and never as moieties covalently conjugated with drugs, even though they are free of the disadvantages of polymers as polydispersity of hyperbranched polymers or laborious syntheses of dendrimers.

**Figure 1:**
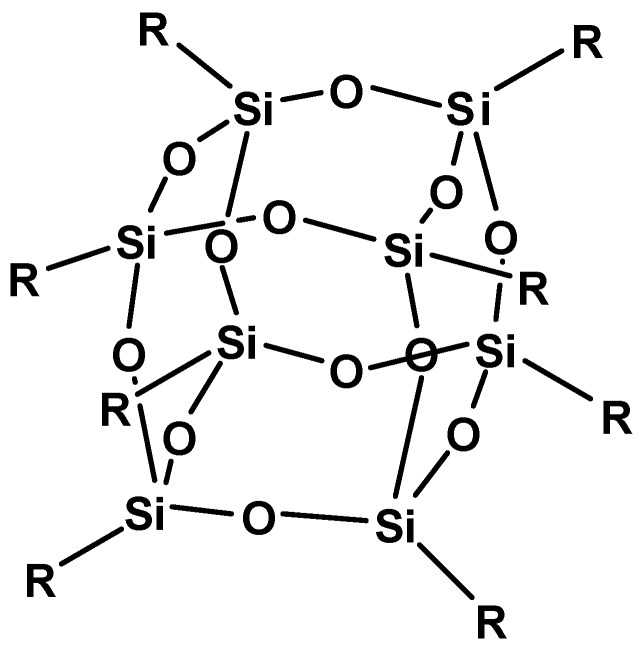
The structure of octaammonium chloride salt of octaaminopropyl polyhedral oligomeric silsesquioxane (oap-POSS), R = -(CH_2_)_3_NH_3_Cl.

The main objective of this work was to study *in vitro* effect caused by polyhedral oligo silsesquioxanes oap-POSS cages (Figure 1). Such cages are characterized by small dimensions (their diameter is approximately 1.5 nm) and are considered as a nanomodel of silica. The long-term goals include using POSS as drug carriers in a targeted anti-cancer therapy. Selective delivery of such systems might be achieved by attaching both anticancer drugs and folic acid residues to the same cage. This approach was never investigated in the case of POSS although was widely explored for other nanosystems.

In order to make an important step towards a new targeted drug delivery system, more is needed to be known about the impact of silsesquioxanes on a cell condition. In the literature, there is a lack of data concerning *in vitro* studies of POSS. Therefore, we undertook toxicity evaluation of POSS towards two cell lines: mouse neuroblastoma (N2a) and embryonic mouse hippocampal cells (mHippoE-18). The choice of these cell lines comes from our current studies on POSS-anticancer drugs nanocomposites. A detailed analysis was performed that included POSS impact on: a cell cycle, cell viability, amount of apoptotic and necrotic cells, and generation of free radical species.

## 2. Results and Discussion

**Figure 2:**
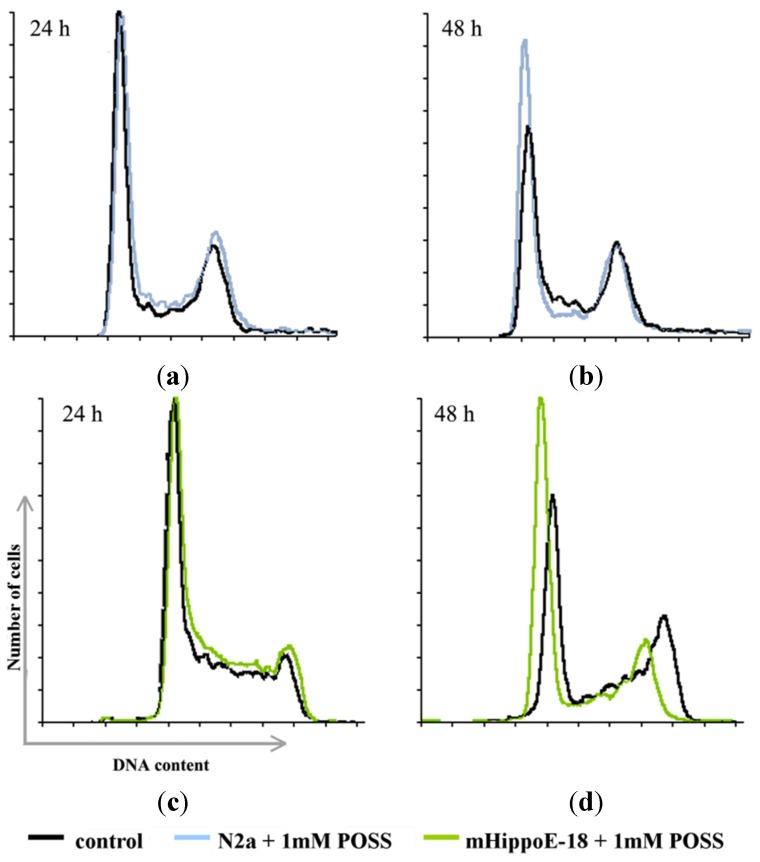
Cell population histograms for N2a cells (**a**,**b**) and mHippoE-18 cells (**c**,**d**) that were untreated (control) and upon incubation with 1 mM POSS for 24 h (**a**,**c**) and 48 h (**b**,**d**).

Cells at different stages of the cell cycle can be distinguished by their DNA content. Experimentally it is achieved by incubation of cells with a fluorescent dye—propidium iodide—that binds to DNA, followed by analysis of the fluorescence intensity of individual cells in a flow cytometer and thereby distinguishing cells in the G_0_/G_1_, S, and M/G_2_ phases of the cell cycle [10]. N2a and mHippoE-18 cells were used to analyze cell cycle kinetics following treatment with POSS that lasted either 24 h or 48 h. For the control samples of both cell lines (Figure 2, Table 1), over half of the cell population was in the G_0_/G_1_ phase. There were also distinct populations of cells in the M/G_2_ phase (almost 30%) and the S phase (less than 15%). In the case of mHippo-E cells, incubation with POSS for 24 h did not cause a statistically significant effect, whereas a twofold increase of the incubation time caused a shift in the cell population from the S and M/G_2_ phases to the G_0_/G_1_ phase. N2a cells were more susceptible to POSS and the same trend was observed even after the 24-hour incubation. After 48 h the effect was more pronounced.

**Table 1:** Cell cycle analysis for control untreated cells and cells treated with 1 mM POSS. Each result represents a mean and SD taken from n ≥ 6 wells from 3 independent experiments. (*) p < 0.05; (**) p < 0.001.

N2a		G_0_/G_1_	S	M/G_2_
24 h	Control	52.70 ± 0.52	13.23 ± 0.93	29.77 ± 0.80
	1 mM POSS	54.57 * ± 0.49	11.40 * ± 0.50	29.53 ± 0.61
48 h	Control	53.67 ± 0.65	13.57 ± 0.70	30.40 ± 1.36
	1 mM POSS	62.63 ** ± 0.91	11.57 ± 0.90	22.83 ** ± 0.17
**mHippoE-18**		**G_0_/G1**	**S**	**M/G2**
24 h	Control	54.17 ± 0.97	12.57 ± 0.68	29.13 ± 0.40
	1 mM POSS	52.83 ± 0.49	11.83 ± 0.95	30.77 ± 0.40
48 h	Control	48.50 ± 0.87	13.33 ± 0.15	28.93 ± 1.10
	1 mM POSS	57.17 ** ± 0.29	9.67 ** ± 0.55	26.23 ** ± 0.60

**Figure 3:**
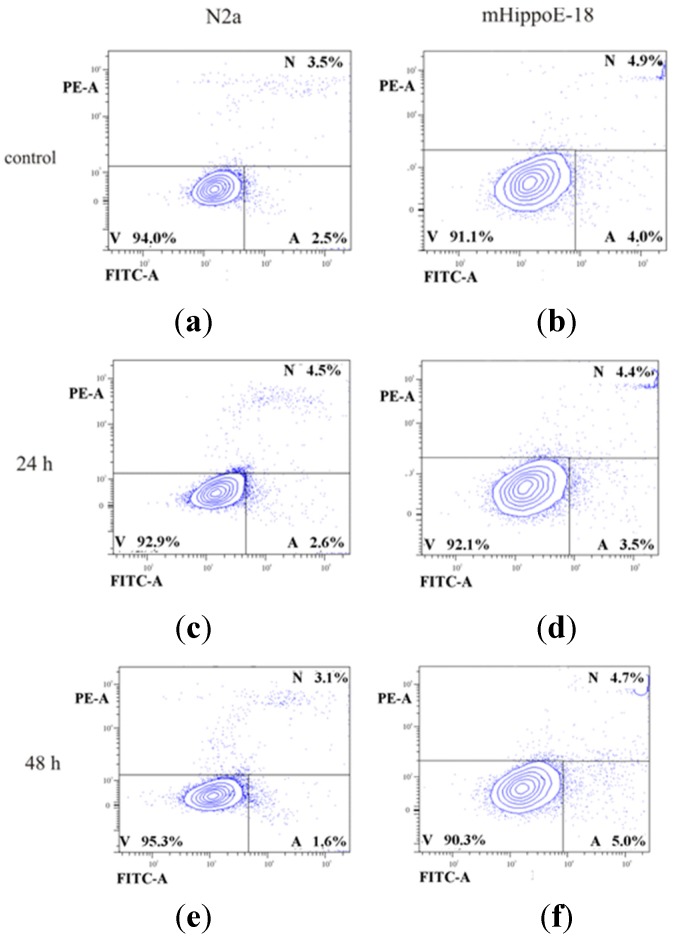
Flow cytometry analysis of the ability of POSS to induce necrosis or apoptosis assayed by Annexin V-FITC and PI fluorescence (V—viable cells; A—apoptotic cells; N—necrotic cells; FITC-A—fluorescence of Annexin V; PE-A—fluorescence of PI). Fractions of apoptotic and necrotic cells after 24 h and 48 h incubation with POSS in a concentration of 1 mM are presented for N2a cells (**a**,**c**,**e**) and mHippoE-18 cells (**b**,**d**,**f**).

Cell cycle analysis is a very complex process since several groups of proteins are involved in its regulation. To verify whether the observed cell arrest in the G_0_/G_1_ phase was a sign of a toxic effect of POSS, we checked the amount of apoptotic and necrotic cells by a double staining method with Annexin V-FITC and propidium iodide. We observed no statistically significant changes in the level of apoptotic, necrotic, and viable cells (Figure 3).

There were more than 90% viable cells after 48 h incubation with POSS for both cell lines. This result was further conformed by an MTT assay (Figure S1 in Supplementary Material).

Recently, it has become clear that reactive oxygen species (ROS) control the cell cycle by influencing the activity of enzymes involved in cell cycle regulation [11,12]. On the other hand, it is known that some nanosystems, such as dendrimers or nanoparticles, are able to generate ROS [13,14]. Therefore, we checked whether POSS could produce ROS. It was determined by using H_2_DCF-DA that is suitable for the estimation of total oxidative activity in living cells. For both tested cell lines we found no ROS generation upon addition of POSS neither after 24 h incubation nor after 48 h incubation (Figure S2 in Supplementary Material).

## 3. Experimental Section

### 3.1. Materials

POSS was made as described in [15] from (3-aminopropyl) triethoxysilane (Sigma Aldrich, Poznan, Poland). All cell culture reagents were purchased from Gibco (Darmstadt, Germany). Flasks and multiwell plates were obtained from Nunc (Darmstadt, Germany). MTT (3-[4,5-dimethylthiazol-2-yl]-2,5-diphenyltetrazolium bromide), PI (propidium iodide) and deoxyribonuclease-free ribonuclease A were purchased from Sigma Aldrich (Poznan, Poland). Trypan blue, H_2_DCF-DA (2’,7’-dichlorodihydrofluorescein), AnnexinV were purchased from BD Biosciences (Franklin Lakes, NJ, USA). Embryonic mouse hippocampal cell line (mHippoE-18) was purchased in CEDARLANE Laboratories Limited (Burlington, ON, Canada). Mouse neuroblastoma cell line (N2a) was purchased from Banca Biologica e Cell Factory (Genova, Italy).

### 3.2. Cell Culture

Mouse neuroblastoma (N2a) and embryonic mouse hippocampal cells (mHippoE-18) were grown in DMEM medium supplemented with 2 mM glutamine and 10% (v/v) fetal bovine serum (FBS). Cells were cultured in T-25 culture flasks at 37 °C in humidified atmosphere containing 5% CO_2_ and subcultured every 2 or 3 days. Cells were harvested and used in experiments after obtaining 80%–90% confluence. The number of viable cells was determined by the trypan blue exclusion assay using Countess Automated Cell Counter (Invitrogen, Waltham, CT, USA). Cells were seeded either in a flat bottom 96-well plates at a density of 1.0 × 10^4^ cells/well in 100 μL of an appropriate medium or in a flat bottom 24-well plates at a density of 1.0 × 10^5^ cells/well in 1 mL of an appropriate medium. After seeding, plates were incubated for 24 h in order to attach cell to the plates.

### 3.3. Cell Cycle Analysis

A cell cycle was analysed using fluorescence of propidium iodide (PI), which binds to DNA. Fluorescence intensity of PI is directly proportional to DNA content [16]. Cells were seeded in 24-well plates at a density of 1.0 × 10^5^ cells/well and incubated with POSS for 24 h and 48 h at 37 °C in humidified atmosphere containing 5% CO_2_. Next, cells were washed with phosphate buffered saline (PBS) and fixed with 96% frozen ethanol before being placed in a freezer for 24 h. Cells collected by centrifugation were washed twice with PBS and resuspended in PBS containing 10 mM Tris HCI (pH 7.5), 5 mM MgCl_2_, 10 μg/mL PI and 10 μg/mL deoxyribonuclease-free ribonuclease A. The cell suspension was incubated in a dark room at 4 °C for 20 min and the fluorescence was measured using a BD Bioscences (Franklin Lakes, NJ, USA) LSR II flow cytometer.

### 3.4. In Vitro Cytotoxicity

The influence of POSS on the cell viability was determined using an MTT assay [17]. Briefly, to the 96-well plates containing cells at the density of 1.0 × 10^4^ cells/well different concentrations of POSS were added. Cells were incubated with POSS for 24 h and 48 h. After the incubation period cells were washed with PBS. Next, 50 μL of a 0.5 mg/mL solution of MTT in PBS was added to each well and cells were further incubated under normal culture conditions for 4 h. After incubation the residue MTT solution was removed and the obtained formazan precipitate was dissolved in dimethyl sulfoxide (DMSO) (100 μL/well). The conversion of the tetrazolium salt (MTT) to a colored formazan by mitochondrial and cytosolic dehydrogenases is a marker of cell viability. Before the absorbance measurement plates were shaken for 1 min and the absorbance at 570 nm was measured with the PowerWave HT Microplate Spectrophotometer (BioTek, Winooski, VT, USA).

### 3.5. Detection of Apoptotic and Necrotic Cells

Detection of apoptotic and necrotic cells was performed by Annexin V and propidium iodide (PI) staining. Annexin V is a protein that specifically binds to phosphatidylserine-phospholipid translocated from the inner layer of the plasma membrane to the outer layer in early apoptotic cells. The cell membrane of apoptotic cells stained with Annexin V conjugated with fluorescein isothiocyanate (FITC) is impermeable to the red fluorescent dye PI, which is able to penetrate the interior of necrotic cells. Therefore, this method is suitable to differentiate between intact, apoptotic and necrotic cell populations. Viable cells show a low level of green fluorescence, while apoptotic cells exhibit an increased level of green fluorescence. Necrotic cells reveal both, red and green fluorescence [18]. Cells were seeded in 24-well plates at the density of 1.0 × 10^5^ cells/well and incubated with silsesquioxane for 24 h and 48 h, as in the previous experiments. Next, cells were washed with PBS, suspended in 500 μL of binding buffer (delivered from the producer) and a mixture consisting of 5 μL of Annexin V fluorescein isothiocyanate (FITC) and 5 μL of PI was added to cell suspension. Samples were incubated at room temperature for 20 min in dark. Fluorescence intensity was measured with a Becton Dickinson LSR II flow cytometer. For control purposes the apoptotic and necrotic cells were used. Apoptosis was induced by camptothecin (80 μM) and necrosis was induced by pentachlorophenol (0.6 μM) (data not shown).

### 3.6. Detection of Intracellular ROS Formation

The intracellular reactive oxygen species (ROS) level is an important marker of oxidative stress. Relative ROS production was determined by using 2’,7’-dichlorodihydrofluorescein (H_2_DCF-DA) that is suitable for the estimation of total oxidative activity in living cells. The detection is based upon the formation of fluorescent dichlorofluorescein (DCF) from nonfluorescent H_2_DCF-DA. H_2_DCF-DA, while incubated with cells, is cleaved by cytosolic esterases to H_2_DCF. This prevents the back-diffusion of the dye to the extracellular space. The product of oxidation of nonfluorescent H_2_DCF is highly fluorescent DCF. Observed fluorescence is proportional to the concentration of intracellular reactive oxygen species [19]. Briefly, to the 24-well plates containing cells at the density of 1.0 × 10^5^ cells/well POSS in a concentration of 1 mM was added. Cells were incubated with POSS for 24 h and 48 h at 37 °C in humidified atmosphere containing 5% CO_2_. After the incubation period cells were washed with PBS. Next, a 2.5 μM H_2_DCF-DA solution in PBS was added to each well. After 15 min of incubation in dark the solution of H_2_DCF-DA was removed, cells were washed with PBS and suspended in 500 μL of PBS. The fluorescence of DCF was measured using a Becton Dickinson LSR II flow cytometer.

### 3.7. Statistical Analysis

Data were expressed as a mean ± SD. Analysis of variance (ANOVA) with Tukey *post hoc* test was used for multiple comparisons. Statistics were calculated using Statistica software (StatSoft, Tulsa, OK, USA), and p-values <0.05 were considered significant.

## 4. Conclusions

POSS in a concentration of 1 mM caused accumulation of cells in the G_0_/G_1_ phase. However, the changes affected less than 10% of the cell population. Considering the fact that POSS did not increase the amount of neither apoptotic nor necrotic cells, it seems likely that the cells were temporarily arrested in the G_0_ phase and were still able to divide. This conclusion is supported by the lack of the cytotoxic effect of POSS. Cell viability measured by an MTT assay revealed no changes when compared to control untreated cells. Moreover, POSS did not generate reactive oxygen species. To sum up, it is the first report showing that POSS have no harmful effect on cells. Therefore, POSS can be considered as potential drug delivery system.

## Supplementary Materials

Supplementary materials can be accessed at: http://www.mdpi.com/1996-1944/8/9/6062/s1.
