# Supplementary material for: In Vitro Studies of Polyhedral Oligo Silsesquioxanes: Evidence for Their Low Cytotoxicity
Source: Materials (Basel). 2015 Sep 10;8(9):6062–70. doi: 10.3390/ma8095291 (PMC5512898; doi:10.3390/ma8095291)
Supplement: Supplementary file 1 [file materials-08-05291-s001.pdf]

Supplementary Materials

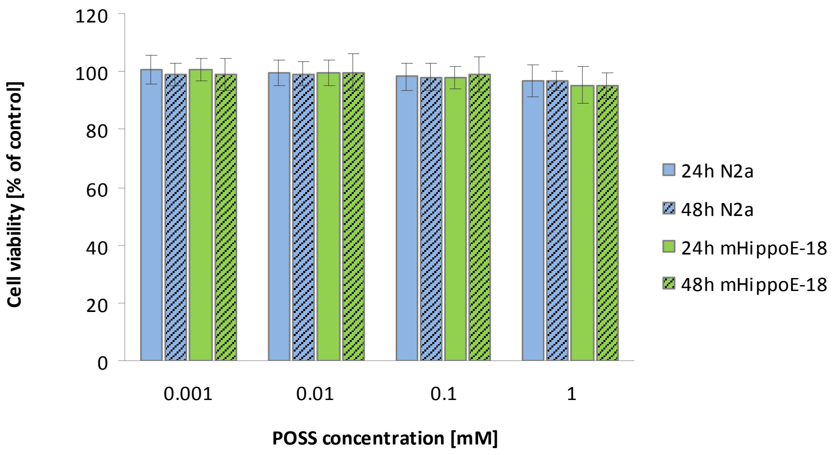

Figure S1. Influence of POSS on viability of N2a mouse neuroblastoma (blue bars) and mHippoE-18 embryonic mouse hippocampal cells (green bars), estimated after 24 h and 48 h of incubation with POSS by MTT assay.

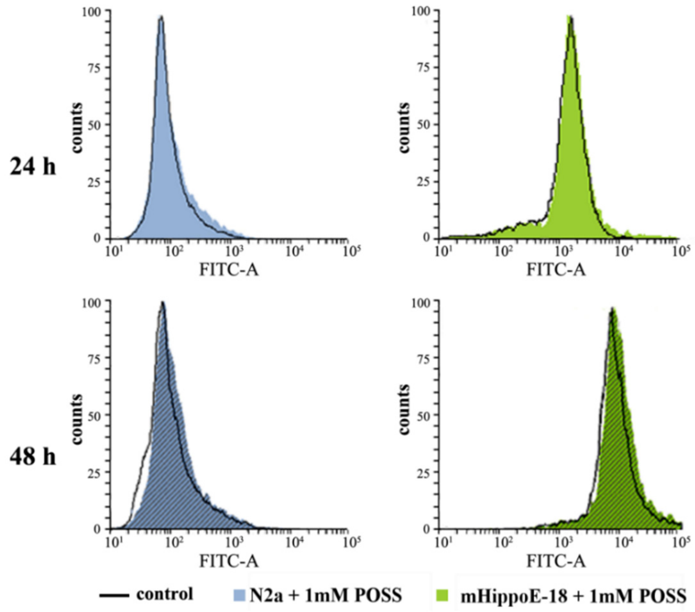

(**a**)

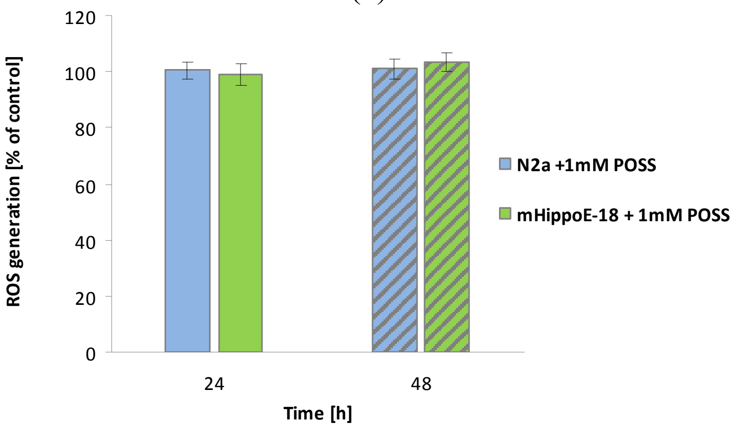

(**b**)

Figure S2. Flow cytometry analysis (**a**) and level of ROS generation in N2a and mHippoE-18 cell lines (**b**) in the presence of 1 mM POSS.
